# Transcriptome analysis reveals the mechanism by which spraying diethyl aminoethyl hexanoate after anthesis regulates wheat grain filling

**DOI:** 10.1186/s12870-019-1925-5

**Published:** 2019-07-19

**Authors:** Daxing Wen, Yan Li, Lifeng He, Chunqing Zhang

**Affiliations:** State Key Laboratory of Crop Biology, Agronomy College, Shandong Agricultural University, Tai’an, Shandong Province 271018 People’s Republic of China

**Keywords:** Diethyl aminoethyl hexanoate, Transcriptome analysis, Grain weight, Seed protein content, Wheat

## Abstract

**Background:**

Diethyl aminoethyl hexanoate (DA-6), a plant growth regulator, has many beneficial effects on agricultural production. DA-6 has been applied to many plant species, but the molecular mechanism by which spraying DA-6 after anthesis regulates wheat grain filling is still unknown.

**Results:**

In this study, we used four DA-6 concentrations: C0 (0 g/L), C2 (2 g/L), C4 (4 g/L), and C6 (6 g/L). The results showed that C4 and C6 led to a significantly higher 1000-grain weight and seed protein content than C0 during two wheat growing seasons. We then subjected samples at 24 days after anthesis (at which point the grain weight increased rapidly) to transcriptome analysis. Flag leaf (L), seed (S), and stem (T) samples under C6 and C0 were used for RNA-seq. The seed samples under C6 compared with C0 (S6vsS0) presented the most differentially expressed genes (DEGs; 2164). Plant hormone signal transduction (*p* = 1.97 × 10^− 4^), protein processing in the endoplasmic reticulum (ER; *p* = 9.04 × 10^− 11^) and starch and sucrose metabolism (*p* = 1.90 × 10^− 10^) pathways were the most markedly enriched pathways in the flag leaves, stems, and seeds, respectively. DEGs involved in sucrose synthesis in the flag leaves, protein processing in ER in the stems, and starch synthesis and protein processing in ER in the seeds were significantly upregulated under C6 compared with C0.

**Conclusions:**

Overall, we propose a model for spraying DA-6 after anthesis to regulate metabolic pathways in wheat, which provides new insights into wheat in response to DA-6.

**Electronic supplementary material:**

The online version of this article (10.1186/s12870-019-1925-5) contains supplementary material, which is available to authorized users.

## Background

Diethyl aminoethyl hexanoate (DA-6), an artificial tertiary amine, is a plant growth regulator that has been applied to many plant species, such as maize, cotton, soybean, peanut, and tomato [1–3]. Extensive studies have demonstrated that DA-6 has many beneficial effects on agricultural production, including increasing the photosynthetic rate and yields, promoting biomass accumulation, and improving germination and seedling establishment of naturally and artificially aged soybean seeds [2, 4]. Moreover, DA-6 can accelerate microalgal growth and enhance the quantity and quality of lipids for biodiesel production [5]. Combining DA-6 and high light can promote astaxanthin accumulation in green microalgae [6]. DA-6 also plays important roles in improving the defence response of plants under diverse environmental stresses, such as salinity stress, chilling stress and heavy metal stress [7–9]. DA-6 can be used to alleviate salinity stress by inducing the advantageous effects of salinity tolerance and decreasing oxidative damage [10]. Foliar sprays of DA-6 can increase cadmium extraction efficiency and can alleviate metal toxicity [11]. DA-6 can also be used in combination with other plant growth regulators and/or phytohormones [9, 12, 13]. Numerous studies have shown the physiological effects of DA-6, but the molecular mechanism remains unknown.

DA-6 has been applied at many stages, including germination, seedling growth, and flowering [1, 2]. The dissipation half-lives of DA-6 are 1.1–2.2 days, 5.4–8.2 days and 1.5–1.9 days in pakchoi, in cotton, and in the soil, respectively [1]. Rapid degradation of DA-6 would be beneficial for the safe use of DA-6 [14]. Spraying DA-6 at the seedling stage can promote plant growth by enhancing photosynthesis and regulating hormone balance in maize and soybean [3]. However, the molecular mechanism by which spraying DA-6 after anthesis influences wheat is still unknown.

Grain weight is a crucial component of grain yield and is significantly influenced by DA-6 levels [2]. Some genes and signalling pathways that determine seed size (grain weight) have been identified, such as the ubiquitin–proteasome pathway, the mitogen-activated protein kinase signalling pathway, genes involved in G-protein signalling and phytohormones and genes that encode transcriptional regulatory factors [15]. DA-6 can increase protein contents in plants [1]. Moreover, genes, environmental conditions, and cultivation patterns also affect seed protein content, which is related to both wheat end use and seed vigour [16–19]. However, the effects of spraying DA-6 after anthesis on wheat grain weight and seed protein content remain unknown.

In this study, the results showed that spraying DA-6 after anthesis increased wheat grain weight and seed protein content. Transcriptome analysis subsequently showed that DA-6 affected plant hormone signal transduction and sucrose synthesis in the flag leaves, protein processing in endoplasmic reticulum (ER) in the stems, and starch synthesis and protein processing in ER in the seeds. Upregulated genes involved in sucrose synthesis in the flag leaves and starch synthesis in the seeds might be associated with increased 1000-grain weight and genes involved in protein processing in ER in both the stems and seeds might contribute to enhanced seed protein content.

## Results

### DA-6 increases wheat grain weight and seed protein content

To investigate the effects of spraying DA-6 after anthesis on wheat grain filling, we tested four DA-6 concentrations: C0 (0 g/L), C2 (2 g/L), C4 (4 g/L), and C6 (6 g/L). The C4 and C6 treatments led to significantly higher 1000-grain weight and seed protein content than did C0 in 2017 and 2018, respectively (Table 1). C6 showed the highest 1000-grain weight during the two wheat growing seasons, which was approximately 10% higher than that of C0. In terms of seed protein content, C6 and C4 displayed the highest seed protein content in 2017 and 2018, respectively. These results suggested that spraying DA-6 could increase grain weight and seed protein content in wheat.

**Table 1:** Effects of spraying DA-6 on the 1000-grain weight and seed protein content in 2017 and 2018

Treatment	1000-grain weight (g)		Seed protein content (%)	
	2017	2018	2017	2018
C0	42.71 c	46.56 c	12.16 b	12.92 b
C2	45.16 b	45.96 c	12.07 b	13.25 a
C4	47.52 a	48.90 b	13.40 a	13.44 a
C6	48.38 a	49.82 a	13.64 a	13.34 a

### Transcriptome analysis of wheat grain filling in response to DA-6 levels

**Fig. 1:**
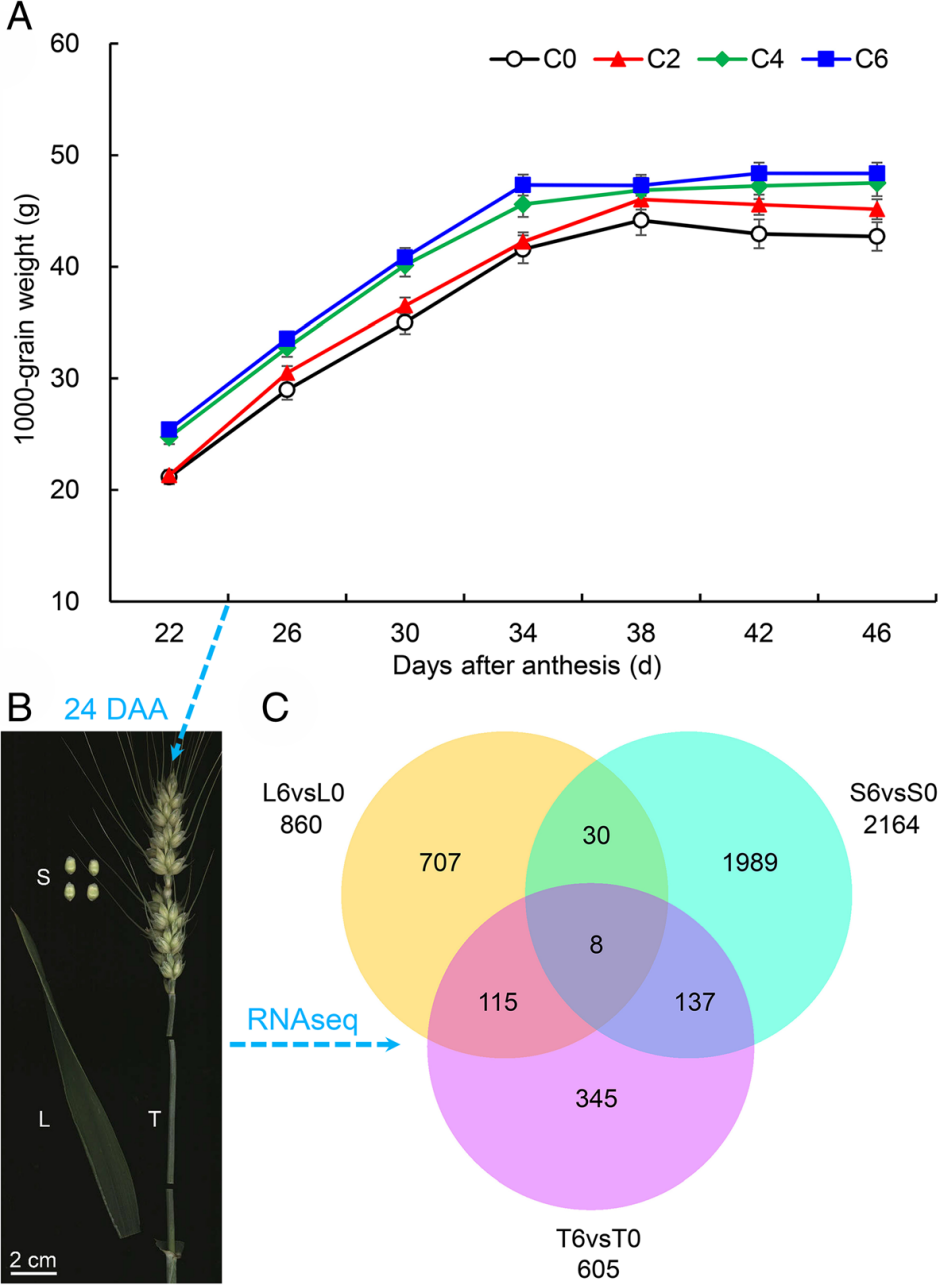
Effects of spraying DA-6 on grain weight accumulation in 2017. We used four DA-6 concentrations: C0 (0 g/L), C2 (2 g/L), C4 (4 g/L), and C6 (6 g/L). **a** 1000-grain weight accumulation; **b** samples at 24 days after anthesis (DAA; at this point, grain weight increased rapidly) for transcriptome analysis. Flag leaf (L), seed (S), and stem (T) samples under C6 and C0 were used for RNA-seq, and three biological replicates were included for each treatment. **c** Venn diagram of differentially expressed genes (DEGs). L6vsL0: flag leaf samples under C6 compared with C0; S6vsS0: seed samples under C6 compared with C0; T6vsT0: stem samples under C6 compared with C0

At the grain-filling stage, C6 showed a higher 1000-grain weight than did C0 from 22 to 46 days after anthesis (Fig. 1a). To explore the molecular mechanism by which spraying DA-6 after anthesis regulates wheat grain filling, we subjected samples at 24 days after anthesis (at which point the grain weight increased rapidly, Fig. 1a) to transcriptome analysis. Flag leaf (L), seed (S), and stem (T) samples under C6 and C0 were used for RNA-seq, and three biological replicates were included for each treatment (Fig. 1b). After removing adapters and sequences with low-quality regions, there remained approximately 36.57–63.60 million clean reads (Additional file 4: Table S1). Approximately 32.49–53.49 million clean reads were then mapped to the wheat genome. These clean reads comprised 4.93–8.46% multiple mapped reads (those mapped to multiple locations) and 70.03–83.89% uniquely mapped reads (those mapped to a single location). Subsequently, we used the DESeq R package (1.18.0) to identify differentially expressed genes (DEGs) using a false discovery rate of < 0.05 as the cutoff. The results showed that 860 DEGs were detected in the flag leaf samples under C6 compared with C0 (L6vsL0), 2164 DEGs were detected in the seed samples under C6 compared with C0 (S6vsS0), and 605 DEGs were detected in the stem samples under C6 compared with C0 (T6vsT0) (Fig. 1c).

**Fig. 2:**
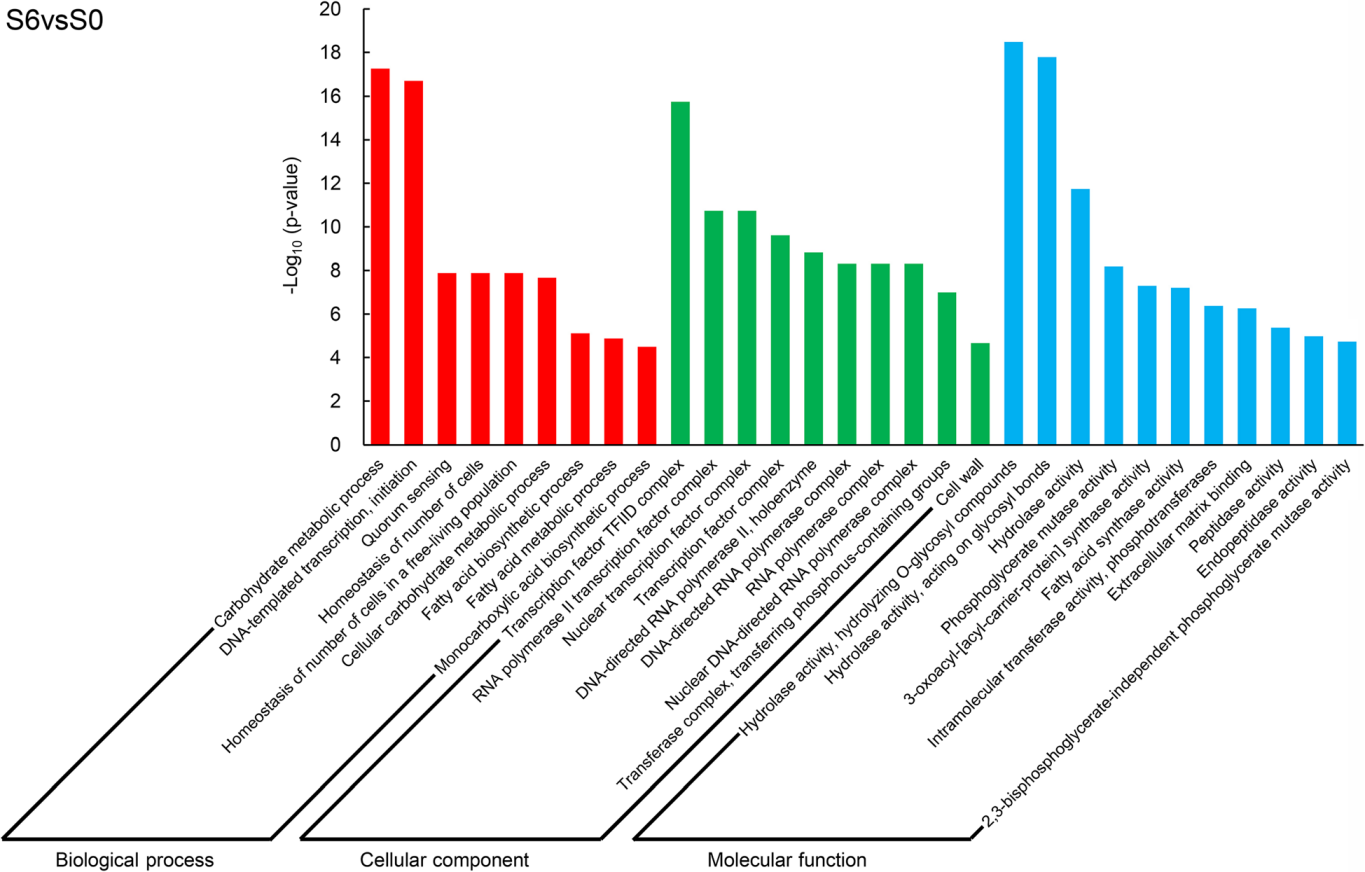
Top 30 significantly enriched GO terms in S6vsS0. S6vsS0: seed samples under C6 compared with C0

To facilitate the global analysis of gene expression, these DEGs were subjected to Gene Ontology (GO) enrichment analysis. In the flag leaf samples (L6vsL0), the most significantly enriched GO terms were protein phosphorylation (GO: 0006468, *p* = 1.49 × 10^− 14^) in the biological process group, anchored component of the plasma membrane (GO: 0046658, *p* = 1.03 × 10^− 3^) in the cellular component group, and protein kinase activity (GO: 0004672, *p* = 1.28 × 10^− 13^) in the molecular function group (Additional file 1: Figure S1). These results indicated that spraying DA-6 after anthesis might regulate protein phosphorylation in flag leaves. Sulfate transport (GO: 0008272, *p* = 6.40 × 10^− 6^) in the biological process group, spindle microtubule (GO: 0005876, *p* = 4.64 × 10^− 2^) in the cellular component, and both extracellular matrix binding (GO: 0050840, *p* = 6.40 × 10^− 6^) and sulfate transmembrane transporter activity (GO: 0015116, *p* = 6.40 × 10^− 6^) in the molecular function group represented the most markedly enriched GO terms in T6vsT0 (Additional file 2: Figure S2). Therefore, sulfate transport and sulfate transmembrane transporter activity in the stems might be involved in the response to DA-6. In S6vsS0, the most prominently enriched GO terms were carbohydrate metabolic process (GO: 0005975, *p* = 5.45 × 10^− 18^) in the biological process group, transcription factor TFIID complex (GO: 0005669, *p* = 1.77 × 10^− 16^) in the cellular component group, and hydrolase activity, hydrolyzing O-glycosyl compounds (GO: 0004553, *p* = 3.27 × 10^− 19^) in the molecular function group (Fig. 2). The results suggested that DA-6 affected carbohydrate metabolism and hydrolase activity in the seeds, which might be related to 1000-grain weight.

### ABA signal transduction and sucrose synthesis in flag leaves are involved in the response to DA-6 levels

**Fig. 3:**
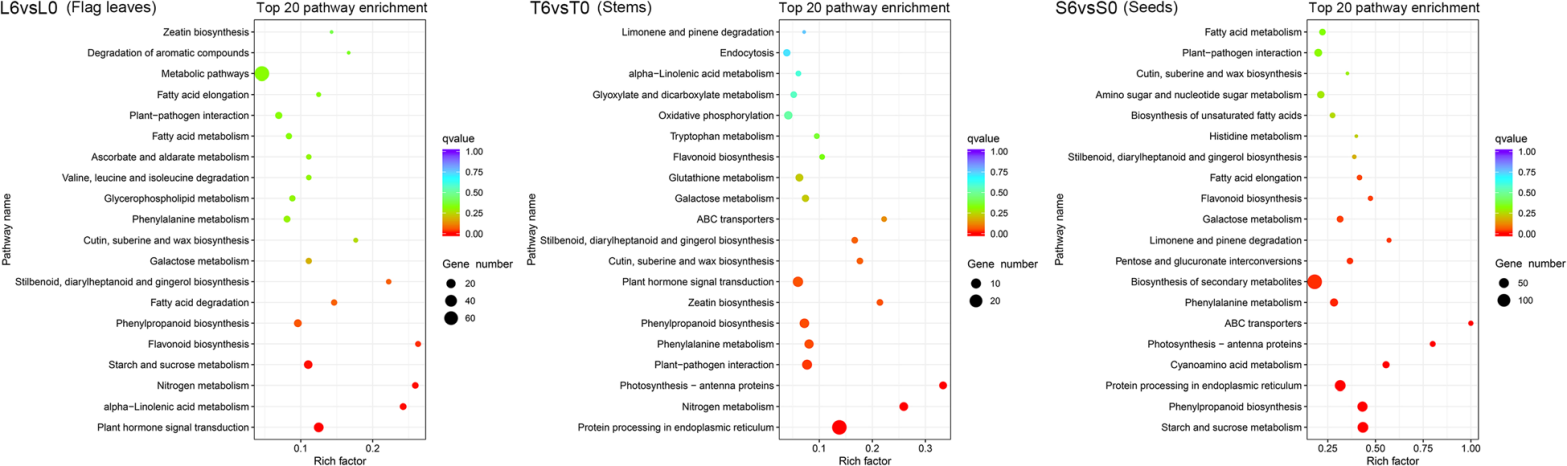
Top 20 KEGG pathways in L6vsL0, T6vsT0, and S6vsS0. L6vsL0: flag leaf samples under C6 compared with C0; T6vsT0: stem samples under C6 compared with C0; S6vsS0: seed samples under C6 compared with C0

**Fig. 4:**
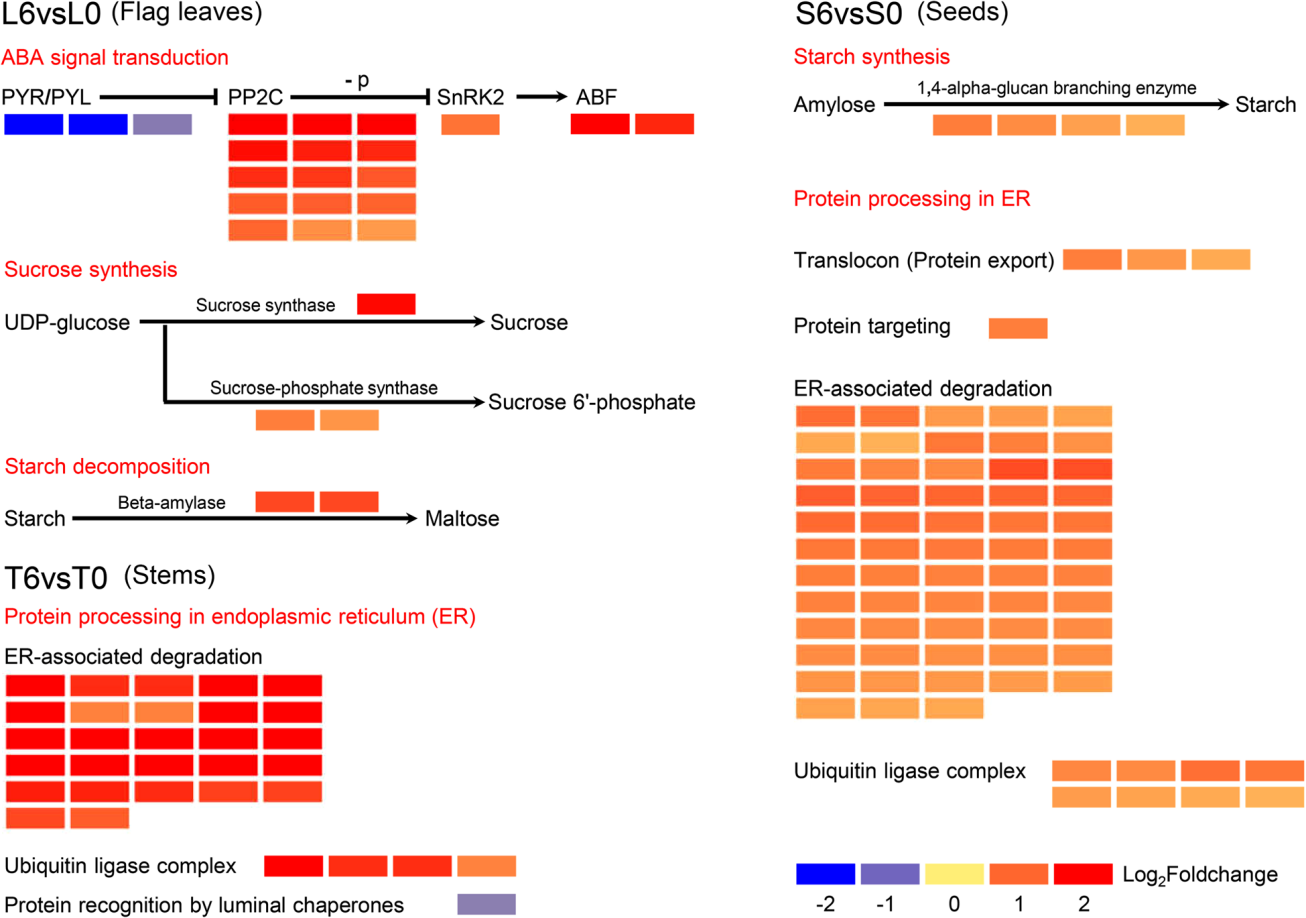
Heat map of selected DEGs enriched in KEGG pathways in L6vsL0, T6vsT0, and S6vsS0. Detailed lists of the DEGs are shown in Additional file 5 Table S2, Additional file 6 Table S3 and Additional file 7 Table S4, respectively. L6vsL0: flag leaf samples under C6 compared with C0; T6vsT0: stem samples under C6 compared with C0; S6vsS0: seed samples under C6 compared with C0. The colour code from blue to red indicates the expression level of the DEGs normalized as the log_2_(fold change). PYR/PYL: ABA receptor PYR/PYL family member; PP2C: protein phosphatase 2C; SnRK2: serine/threonine-protein kinase SRK2; ABF: ABA-responsive element binding factor

To identify the metabolic pathways involved in response to DA-6 levels, we further analysed the Kyoto Encyclopedia of Genes and Genomes (KEGG) enrichment pathways. Many metabolic pathways were enriched in the flag leaves, stems, and seeds (Fig. 3). Plant hormone signal transduction was the most significantly enriched pathway in L6vsL0, followed by alpha-linolenic acid metabolism, nitrogen metabolism, starch and sucrose metabolism, and flavonoid biosynthesis. ABA signal transduction represented the largest group within plant hormone signal transduction. Abscisic acid receptor PYR/PYL family genes (*TraesCS4A02G114400*, *TraesCS4B02G189800* and *TraesCS2D02G087500*) were dramatically downregulated, while protein phosphatase 2C, serine/threonine-protein kinase SRK2 and ABA-responsive element binding factor-related genes were notably upregulated in L6vsL0 (Fig. 4, Additional file 5: Table S2). Moreover, three DEGs (*TraesCS4B02G167500*, *TraesCS3A02G015500* and *TraesCSU02G044500*) involved in sucrose synthesis and two DEGs (*TraesCS5A02G513200* and *TraesCS4B02G344300*) related to starch decomposition were upregulated in the starch and sucrose metabolism pathway (Fig. 4, Additional file 5: Table S2). Sucrose synthase- and sucrose-phosphate synthase-related genes were upregulated in L6vsL0. The results suggested that DA-6 might affect ABA signal transduction, sucrose synthesis and starch decomposition in flag leaves at the grain-filling stage.

### Protein processing in ER in the stems are involved in the response to DA-6 levels

In T6vsT0, the most significantly enriched pathway was protein processing in ER, followed by nitrogen metabolism, photosynthesis - antenna proteins, plant-pathogen interactions, phenylalanine metabolism and phenylpropanoid biosynthesis (Fig. 3). Except for one downregulated gene related to protein recognition by luminal chaperones, genes involved in ER-associated degradation and the ubiquitin ligase complex were markedly upregulated in protein processing in ER (Fig. 4, Additional file 6: Table S3). Many genes involving the heat shock 70 kDa protein, molecular chaperone HtpG, DnaJ homolog subfamily A member 2 and HSP20 family protein were upregulated in ER-associated degradation (Additional file 6: Table S3). In terms of the ubiquitin ligase complex, there were four upregulated genes. Only one downregulated gene was related to protein recognition by luminal chaperones. These results indicated that DA-6 might have influenced protein processing in ER in the stem.

### Starch synthesis and protein processing in ER in the seeds are involved in the response to DA-6 levels

Compared with samples from the flag leaves and stems, the seed samples showed more significantly enriched pathways. Starch and sucrose metabolism was the most significantly enriched pathway in S6vsS0, followed by phenylpropanoid biosynthesis, protein processing in ER, cyanoamino acid metabolism, photosynthesis - antenna proteins, ABC transporters, phenylalanine metabolism, biosynthesis of secondary metabolites, pentose and glucuronate interconversion, limonene and pinene degradation, galactose metabolism, flavonoid biosynthesis and fatty acid elongation (Fig. 3). In starch and sucrose metabolism, four 1,4-alpha-glucan branching enzyme genes (*TraesCS7A02G549100*, *TraesCS7D02G535400*, *TraesCS7D02G535500* and *TraesCS7B02G472500*), which are involved in starch synthesis, were upregulated (Fig. 4, Additional file 7: Table S4). This phenomenon might be one of the reasons that the C6 treatment showed a higher 1000-grain weight than did C0. S6vsS0 had more genes enriched in protein processing in ER than did T6vsT0. All genes enriched in protein processing in ER in S6vsS0 were upregulated. In addition to the genes involved in ER-associated degradation and the ubiquitin ligase complex, translocon (protein export)- and protein targeting-related genes also were upregulated in S6vsS0. Many genes involving the heat shock 70 kDa protein, molecular chaperone HtpG, HSP20 family proteins and ubiquitin conjugation factor E4 were upregulated in ER-associated degradation in S6vsS0 (Additional file 7: Table S4). The number of genes enriched in the ubiquitin ligase complex in S6vsS0 was twice that in T6vsT0. These results suggested that protein processing in ER in the seeds was involved in the response to DA-6 levels, which might be related to seed protein content.

### Validation of RNA-Seq data by qRT-PCR

**Fig. 5:**
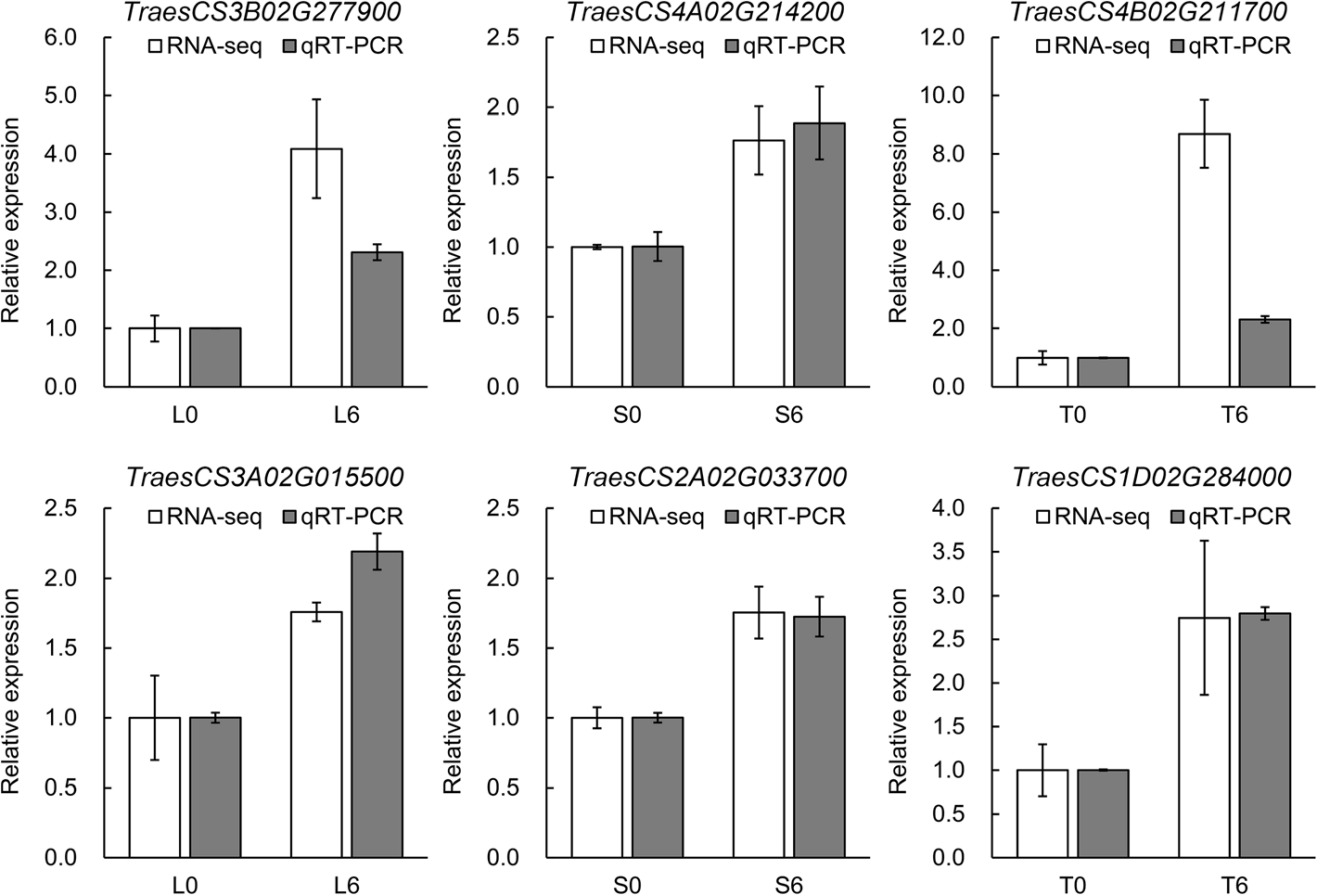
Validation of DEGs by qRT-PCR. The white bar and grey bar represent the fold change of the relative expression level from the RNA-seq data and qRT-PCR data, respectively. The wheat *Actin* gene, as an internal control, was used to normalize the expression levels of the target genes. The error bars represent the standard deviations of three replicates. L6vsL0: flag leaf samples under C6 compared with C0; T6vsT0: stem samples under C6 compared with C0; S6vsS0: seed samples under C6 compared with C0

To validate the RNA-seq results, we randomly selected six DEGs for qRT-PCR assays. Among the DEGs, two were upregulated in L6vsL0, S6vsS0 and T6vsT0 (Fig. 5). All six DEGs in the qRT-PCR assays were essentially consistent with their transcript abundance changes identified by RNA-seq, suggesting the transcriptome data were reliable. Although the fold change of *TraesCS4B02G211700* according to qRT-PCR was lower than that in RNA-seq data, *TraesCS4B02G211700* was upregulated in both the qRT-PCR and RNA-seq data.

## Discussion

Extensive studies have shown that DA-6 can be applied at different stages and can affect various aspects of plants [1–3]. However, the mechanism of wheat at the grain-filling stage in response to DA-6 is still unknown. In this study, spraying DA-6 one week after anthesis was used to study the effects of DA-6 on grain filling. DA-6 treatments at the V_3_ stage affect many aspects of corn and soybean seedlings, such as increasing plant height and leaf area, improving the root-to-shoot ratio, and enhancing photosynthesis [3]. Therefore, spraying DA-6 before anthesis or at the seedling stage might affect several different aspects (such as plant height, flowering and seed number per spike) in addition to grain weight. Moreover, the optimal dose of DA-6 at the seedling stage might be lower than that in this study because the biomass per square metre at the seedling stage was far less than that at anthesis. Grain weight is one of the components that determines wheat grain yield. The spike number per hectare and seed number per spike were similar among C0, C2, C4, and C6. Therefore, only seed weight determined grain yield in this study. From the perspective of grain yield, the optimal stage for spraying DA-6 needs further study.

DA-6 is a plant growth regulator, so an understanding of its receptor and the relationship between DA-6 and plant hormones will help us further understand its action [4]. In this study, the plant hormone signal transduction pathway was the most significantly enriched pathway in the flag leaves. In addition to most of the DEGs involved in ABA signal transduction, one DEG (*TraesCS6B02G027800*, log_2_(fold change) = 1.6506) involved in cytokinin signal transduction and one DEG (*TraesCS2A02G099900*, log_2_(fold change) = − 0.7537) involved in ethylene signal transduction were also enriched in the plant hormone signal transduction pathway. Although the plant hormone signal transduction pathway was not significantly enriched in T6vsT0 and S6vsS0, there were more than ten DEGs involved in several plant hormone signal transduction pathways in T6vsT0 and S6vsS0. Plant hormone signal transduction was different among the flag leaves, seeds, and stems in response to DA-6. ABA and cytokinine signal transduction involved DEGs in L6vsL0, T6vsT0, and S6vsS0, and ABA signal transduction revealed more DEGs than did cytokinin signal transduction. Moreover, three DEGs (*TraesCS4A02G114400*, log_2_(fold change) = − 2.6814; *TraesCS4B02G189800*, log_2_(fold change) = − 2.0476; *TraesCS2D02G087500*, log_2_(fold change) = − 0.84083) in L6vsL0 and two DEGs (*TraesCS4A02G114400*, log_2_(fold change) = − 1.9287; *TraesCS4D02G191200*, log_2_(fold change) = − 1.6032) in T6vsT0 were implicated in the ABA receptor PYR/PYL family. All five DEGs were markedly downregulated. Therefore, in terms of plant hormones, spraying DA-6 after anthesis in wheat might mainly regulate the ABA signal transduction pathway.

Sucrose is the main product of photosynthesis in wheat, and starch and protein are the major storage nutrients in wheat seed. A previous study showed that sucrose and starch contents in soybean leaves and pods increased from flowering to podding in response to spraying DA-6 at the seedling stage [2]. In this study, spraying DA-6 after anthesis stimulated starch and sucrose metabolism in the flag leaves and seeds. Sucrose synthesis-related DEGs were upregulated in L6vsL0, and starch synthesis-related DEGs were upregulated in S6vsS0. DA-6 increased not only sucrose synthesis in the flag leaves (source organs) but also starch synthesis in the seeds (sink organs), which indicated that DA-6 might enhance the synthesis ability of sucrose and starch. Therefore, the 1000-grain weight under C4 and C6 was significantly higher than that under C0 during two wheat growing seasons.

Seed protein content affects not only seed processing quality but also seed vigour [18, 19]. Regardless of the presence or absence of salt stress, DA-6 treatment significantly increased the concentrations of soluble sugars and soluble protein in *Cassia obtusifolia* L. [10]. In this study, C4 and C6 had markedly higher seed protein content than did C0 during two wheat growing seasons. KEGG enrichment analysis showed that protein processing in ER pathway was significantly enriched in both T6vsT0 and S6vsS0. Moreover, DEGs involved in protein processing in ER pathway were mainly involved in ER-associated degradation and the ubiquitin ligase complex in T6vsT0 and S6vsS0. With respect to ER-associated degradation and the ubiquitin ligase complex, the expression of DEGs in T6vsT0 had a higher log_2_(fold change) than that in S6vsS0, but S6vsS0 had more DEGs than did T6vsT0. In addition to these DEGs, three other DEGs involved in protein export were also significantly upregulated in S6vsS0. These three DEGs might be associated with protein export from the ER to storage locations. Therefore, the results suggested that DA-6 regulation of protein processing in ER in the stems and seeds might be related to enhanced seed protein content.

**Fig. 6:**
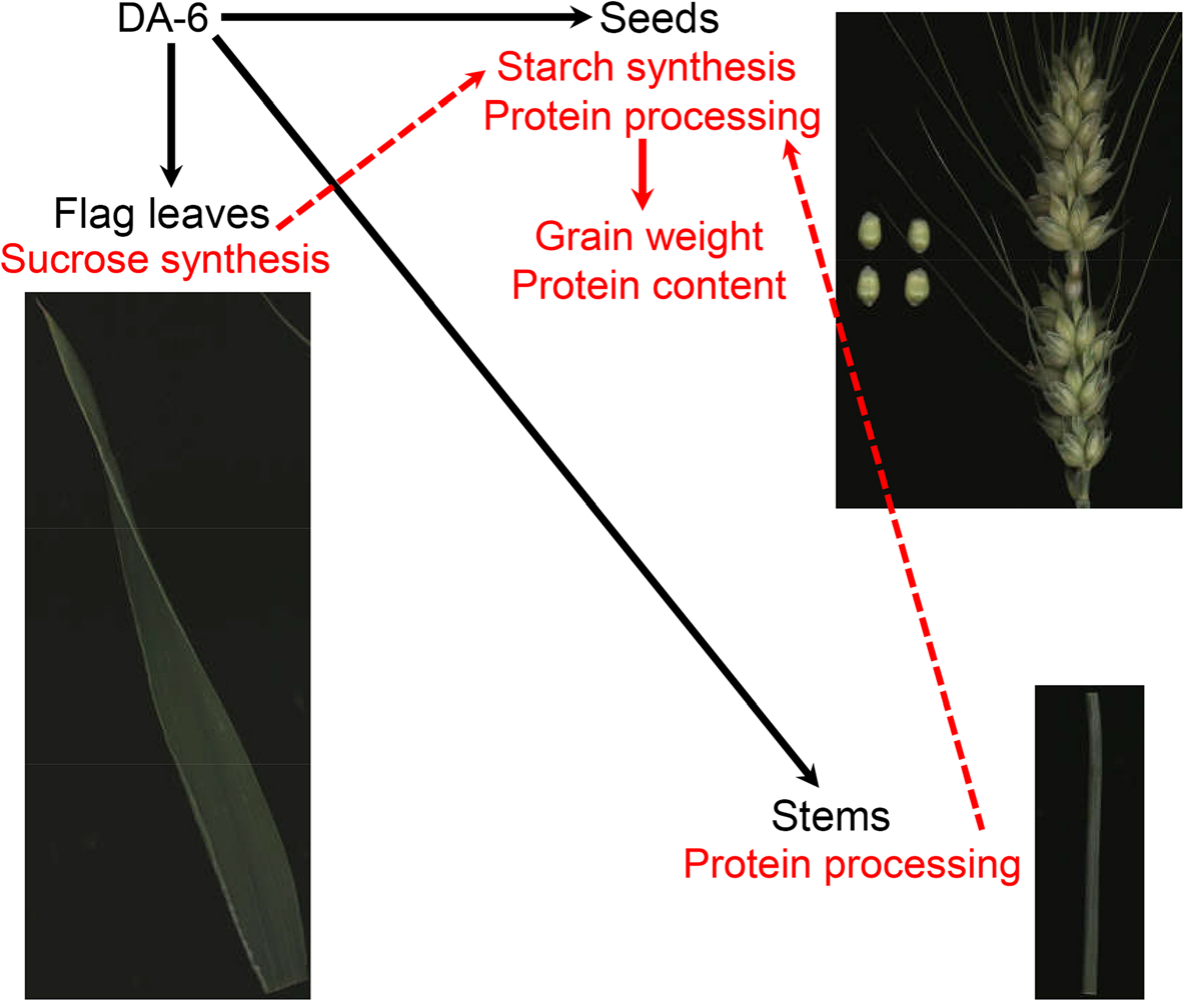
Possible network by which DA-6 regulates the metabolic pathways in flag leaves, stems, and seeds of wheat. The solid lines indicate definite regulation; the dotted lines indicate conjectural regulation

Overall, we propose a model for wheat in response to spraying DA-6 after anthesis (Fig. 6). DA-6 upregulates the expression level of genes involved in sucrose synthesis in the flag leaves, protein processing in ER in the stems, and starch synthesis and protein processing in ER in the seeds. Moreover, sucrose synthesis in the flag leaves might promote starch synthesis in the seeds, and protein processing in ER in the stems might enhance protein processing in ER in the seeds. The increase in starch synthesis and protein processing in ER in the seed enhances grain yield and seed protein content.

## Conclusion

Spraying an appropriate amount of DA-6 at one week after anthesis can increase wheat (Shannong 23) grain yields and seed protein content. Transcriptome analysis revealed that genes involved in sucrose synthesis in the flag leaves, protein processing in ER in the stems, and starch synthesis and protein processing in ER in the seeds were significantly upregulated. Therefore, this study provides new insights into wheat in response to DA-6.

## Methods

### Material and experimental design

Wheat cultivar Shannong 23, which is extensively cultivated in Shandong Province, was used as the experimental material. The seeds were acquired from Shandong Agricultural University. Field experiments were conducted at the experimental station of Shandong Agricultural University (36°17′N and 117°2′E, Tai’an city, Shandong Province, China) during the wheat growing seasons (October–June) in 2016–2017 and 2017–2018. Field experiments were laid out in a randomized block design. Each field plot was 2 m × 2 m, and the soil type was a sandy loam. Nitrogen, phosphate, and potash fertilizers were applied as described previously [20]. The monthly rainfall and mean temperature during the two wheat growing seasons are shown in Additional file 3: Figure S3. The irrigation amount under all treatments was equal, and no water stress occurred. DA-6 powder (purity: 98.5%, Maosheng Chemical Plant, Changzhou, China) was dissolved in distilled water before spraying. We applied four DA-6 concentrations: C0 (0 g/L), C2 (2 g/L), C4 (4 g/L), and C6 (6 g/L). Moreover, organic silicone (a surfactant) was added to the DA-6 solution at a rate of 1 mL/L. At one week after anthesis (May 2, 2017, and May 3, 2018), the DA-6 solution was sprayed by an electric sprayer with a single nozzle at a rate of 200 mL/m^2^ in the afternoon (between 16:00 and 18:00, approximately 24 °C). The seeds were harvested on June 6, 2017, and June 7, 2018. Diseases, pests, and weeds were well controlled during the two wheat growing seasons.

### Grain weight and seed protein content

A DA7200 device (Perten, Stockholm, Sweden) was used to measure seed moisture contents and seed protein content [19]. One thousand seeds were randomly counted to measure the 1000-grain weight. Each treatment included three replicates. The 1000-grain weight was then adjusted to 13% seed moisture content.

### RNA isolation and quality determination

Samples (flag leaves, four seeds located in the middle part of the spike, and approximately 5 cm long stems below the spikes) of twenty plants were pooled together as one biological sample, and each treatment included three biological replicates. The samples were stored at − 80 °C after being frozen in liquid nitrogen. RNA extraction, quality determination, and RNA concentration measurements were performed according to a previous study, with some modifications [21]. Total RNA was extracted using TRIzol reagent (Invitrogen, ON, Canada) or a DP441 RNA extraction kit (Tiangen, Beijing, China) according to the manufacturers’ protocols. A NanoPhotometer spectrophotometer (IMPLEN, CA, USA) was used to examine RNA purity. Moreover, RNA was monitored on 1% agarose gels. A Qubit RNA Assay Kit in a Qubit 2.0 Fluorometer (Life Technologies, CA, USA) and an RNA Nano 6000 Assay Kit of the Bioanalyzer 2100 system (Agilent Technologies, CA, USA) were used to measure RNA concentrations and evaluate RNA integrity, respectively. High-quality RNA was used for subsequent RNA-seq library construction.

### RNA sequencing and bioinformatics analysis

RNA-seq library construction, RNA-seq, and bioinformatics analysis were performed as described previously [21]. A NEBNext Ultra™ RNA Library Prep Kit for Illumina (NEB, MA, USA) was used for RNA-seq library construction according to the manufacturer’s protocol. Index codes were added to the sequences of different samples. Magnetic beads with oligo-dT were used to enrich mRNA from approximately 3 μg of RNA per sample. Double-stranded cDNAs were synthesized according to the manufacturer’s instructions. cDNA fragments that were 150~200 bp in length were selected by using an AMPure XP system (Beckman Coulter, Beverly, USA). These RNA-seq libraries were sequenced on an Illumina HiSeq platform for generating 150 bp paired-end reads. Reads containing adapters, reads containing poly-N, and low-quality reads were removed from the raw reads. Moreover, the Q20, Q30 and GC contents of the clean data were also calculated. The clean reads were subsequently mapped to the wheat genome sequence (ftp://ftp.ensemblgenomes.org/pub/plants/release-41/fasta/triticum_aestivum/dna/) by using TopHat v2.0.12 [22]. The read numbers of each gene were counted by using HTSeq v0.6.1. The DESeq R package (1.18.0) was used for performing a differential expression analysis of the two groups [23, 24]. Benjamini and Hochberg’s approach was then used to adjust the resulting *p*-values for controlling the false discovery rate. Genes with an adjusted *p*-value < 0.05 were considered differentially expressed genes (DEGs). GO and KEGG enrichment analyses were carried out based on the DEGs. The GO enrichment analysis of DEGs was performed by using the GOseq R package, and GO terms with corrected *p*-values < 0.05 were assigned as significantly enriched GO terms [25]. The KEGG enrichment analysis of the DEGs was performed by using KOBAS software [26]. Similarly, the KEGG pathways with corrected *p*-values < 0.05 were assigned as significantly enriched pathways.

### qRT-PCR

Validation of the gene expression levels revealed by RNA-seq was carried out by qRT-PCR. An ABI StepOne Plus Real-Time PCR System (Applied Biosystems, CA, USA) was used to perform all qRT-PCR analyses. Gene-specific primers for qRT-PCR were synthesized by Sangon Biotech (Shanghai, China) and are listed in Additional file 8: Table S5. A PrimeScript RT reagent kit (Takara, Dalian, China) was used to synthesize cDNA from total RNA. The wheat *Actin* gene, as an internal control, was used to normalize the expression levels of the target genes [27]. Each qRT-PCR experiment was repeated three times, and the mean expression level and standard deviation were calculated using the 2^−△△Ct^ method [28].

### Statistical analysis

One-way analysis of variance and Duncan’s multiple tests were performed by using SPSS 19.0 software (IBM, New York, USA).

## Additional files

Additional file 1: **Figure S1** Top 30 significantly enriched GO terms in L6vsL0. (DOCX 1233 kb)

Additional file 2: **Figure S2** Significantly enriched GO terms in T6vsT0. (DOCX 1122 kb)

Additional file 3: **Figure S3** Meteorological data recorded during the period of wheat growth (October to June). (DOCX 708 kb)

Additional file 4: **Table S1** Summary of RNA-seq data. (DOCX 15 kb)

Additional file 5: **Table S2** List of selected genes for KEGG pathways in L6vsL0. (DOCX 14 kb)

Additional file 6: **Table S3** List of selected genes for KEGG pathways in T6vsT0. (DOCX 15 kb)

Additional file 7: **Table S4** List of selected genes for KEGG pathways in S6vsS0. (DOCX 16 kb)

Additional file 8: **Table S5** Primers used for qRT-PCR. (DOCX 14 kb)

## Data Availability

The datasets generated and/or analysed in this study are included in the article and additional files. The RNA-seq data have been deposited in the NCBI Sequence Read Archive (SRA, http://www.ncbi.nlm.nih.gov/sra) under accession number PRJNA544398.

## Abbreviations

DA-6: Diethyl aminoethyl hexanoate
DEG: differentially expressed gene
ER: endoplasmic reticulum
GO: Gene Ontology
KEGG: Kyoto Encyclopedia of Genes and Genomes
L6vsL0: flag leaf samples under C6 compared with C0
S6vsS0: seed samples under C6 compared with C0
T6vsT0: stem samples under C6 compared with C0
