# Supplementary material for: Transcriptome analysis reveals the mechanism by which spraying diethyl aminoethyl hexanoate after anthesis regulates wheat grain filling
Source: BMC Plant Biol. 2019 Jul 19;19:327. doi: 10.1186/s12870-019-1925-5 (PMC6642493; doi:10.1186/s12870-019-1925-5)
Supplement: Supplementary file 1 — Figure S1 Top 30 significantly enriched GO terms in L6vsL0. (DOCX 1233 kb) [file 12870_2019_1925_MOESM1_ESM.docx]

**
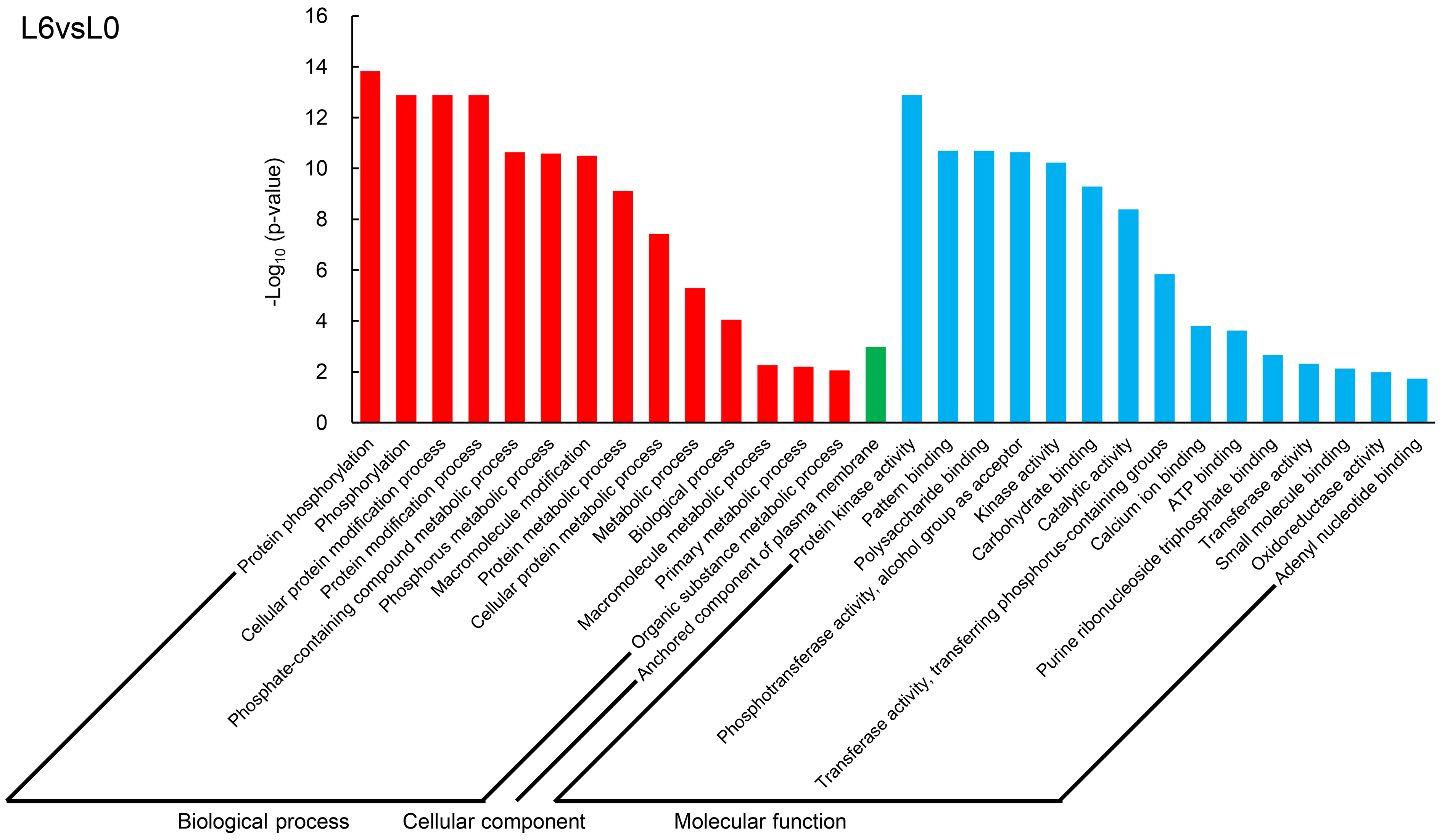
**

**Figure S1.** Top 30 significantly enriched GO terms in L6vsL0. L6vsL0: flag leaf samples under C6 compared with C0.
