# Supplementary figures and images for: Transcriptome analysis reveals the mechanism by which spraying diethyl aminoethyl hexanoate after anthesis regulates wheat grain filling
Source: BMC Plant Biol. 2019 Jul 19;19:327. doi: 10.1186/s12870-019-1925-5 (PMC6642493; doi:10.1186/s12870-019-1925-5)

**
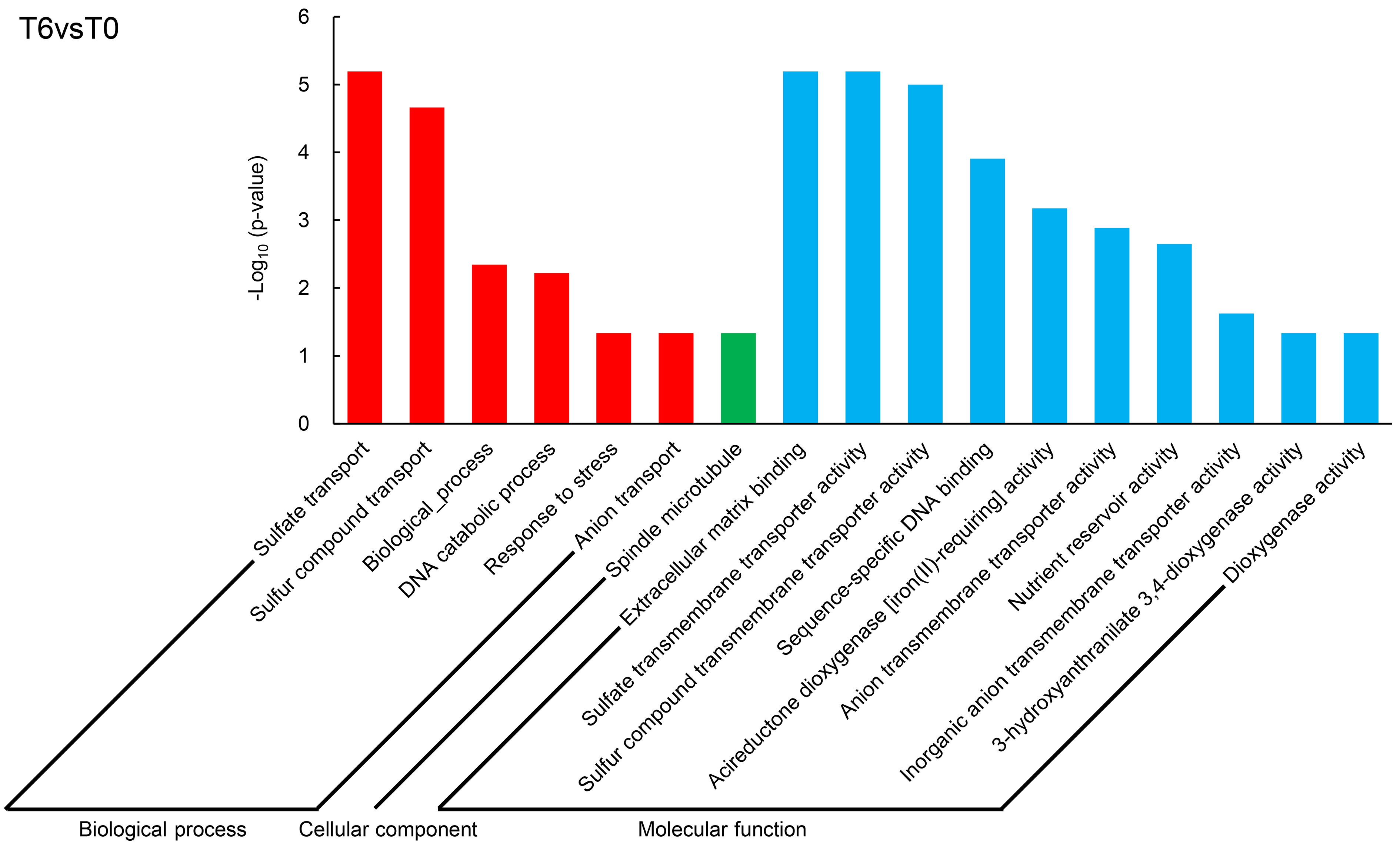
**

**Figure S2.** Significantly enriched GO terms in T6vsT0. T6vsT0: stem samples under C6 compared with C0.

Supplement: Supplementary file 2 — Figure S2 Significantly enriched GO terms in T6vsT0. (DOCX 1122 kb) [file 12870_2019_1925_MOESM2_ESM.docx]

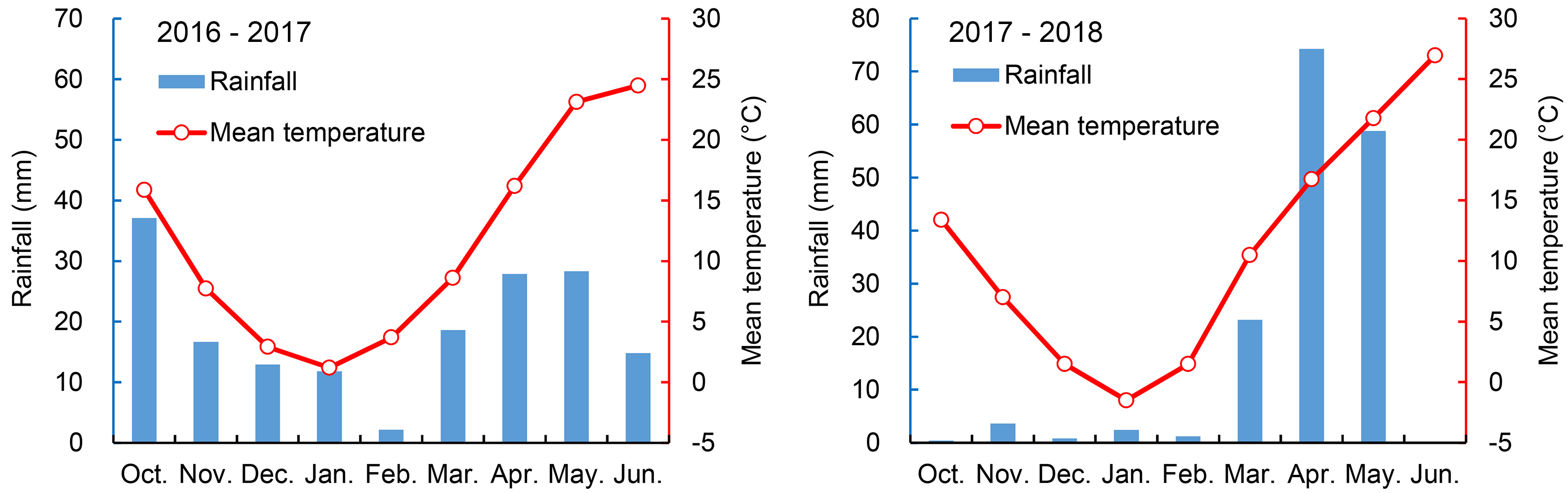


**Figure S3.** Meteorological data recorded during the period of wheat growth (October to June).

Supplement: Supplementary file 3 — Figure S3 Meteorological data recorded during the period of wheat growth (October to June). (DOCX 708 kb) [file 12870_2019_1925_MOESM3_ESM.docx]
