# Supplementary material for: Transcriptome analysis reveals the mechanism by which spraying diethyl aminoethyl hexanoate after anthesis regulates wheat grain filling
Source: BMC Plant Biol. 2019 Jul 19;19:327. doi: 10.1186/s12870-019-1925-5 (PMC6642493; doi:10.1186/s12870-019-1925-5)
Supplement: Supplementary file 4 — Table S1 Summary of RNA-seq data. (DOCX 15 kb) [file 12870_2019_1925_MOESM4_ESM.docx]

**Table S1.** Summary of RNA-seq data

| Sample | Clean reads | Total mapped | Multiple mapped | Uniquely mapped |
| --- | --- | --- | --- | --- |
| L0_1 | 56139384 | 50683141 (90.28%) | 3587982 (6.39%) | 47095159 (83.89%) |
| L0_2 | 60372704 | 53494149 (88.61%) | 3685899 (6.11%) | 49808250 (82.50%) |
| L0_3 | 53174838 | 47433567 (89.20%) | 3397973 (6.39%) | 44035594 (82.81%) |
| L6_1 | 54529520 | 47669646 (87.42%) | 3128745 (5.74%) | 44540901 (81.68%) |
| L6_2 | 48604750 | 43200536 (88.88%) | 2907078 (5.98%) | 40293458 (82.90%) |
| L6_3 | 47823272 | 41731624 (87.26%) | 2704559 (5.66%) | 39027065 (81.61%) |
| S0_1 | 47577156 | 39156938 (82.30%) | 3405209 (7.16%) | 35751729 (75.14%) |
| S0_2 | 51273866 | 42300685 (82.50%) | 3926958 (7.66%) | 38373727 (74.84%) |
| S0_3 | 56012776 | 46852022 (83.65%) | 3883962 (6.93%) | 42968060 (76.71%) |
| S6_1 | 52790504 | 41313469 (78.26%) | 4342023 (8.23%) | 36971446 (70.03%) |
| S6_2 | 56941364 | 45122675 (79.24%) | 4815058 (8.46%) | 40307617 (70.79%) |
| S6_3 | 63602822 | 52039427 (81.82%) | 5175753 (8.14%) | 46863674 (73.68%) |
| T0_1 | 41409952 | 36857805 (89.01%) | 2263702 (5.47%) | 34594103 (83.54%) |
| T0_2 | 36571438 | 32490277 (88.84%) | 2120383 (5.80%) | 30369894 (83.04%) |
| T0_3 | 52942560 | 47834898 (90.35%) | 3241388 (6.12%) | 44593510 (84.23%) |
| T6_1 | 46069354 | 41612122 (90.32%) | 2706121 (5.87%) | 38906001 (84.45%) |
| T6_2 | 37704170 | 33702231 (89.39%) | 1857841 (4.93%) | 31844390 (84.46%) |
| T6_3 | 38845936 | 34962321 (90.00%) | 2210123 (5.69%) | 32752198 (84.31%) |
