# Supplementary material for: Transcriptome analysis reveals the mechanism by which spraying diethyl aminoethyl hexanoate after anthesis regulates wheat grain filling
Source: BMC Plant Biol. 2019 Jul 19;19:327. doi: 10.1186/s12870-019-1925-5 (PMC6642493; doi:10.1186/s12870-019-1925-5)
Supplement: Supplementary file 5 — Table S2 List of selected genes for KEGG pathways in L6vsL0. (DOCX 14 kb) [file 12870_2019_1925_MOESM5_ESM.docx]

**Table S2.** List of selected genes for KEGG pathways in L6vsL0.

|  | Gene ID | KO | Log_2_(fold change) |
| --- | --- | --- | --- |
| ABA signal transduction | TraesCS4A02G114400 | Abscisic acid receptor PYR/PYL family | -2.6814 |
|  | TraesCS4B02G189800 | Abscisic acid receptor PYR/PYL family | -2.0476 |
|  | TraesCS2D02G087500 | Abscisic acid receptor PYR/PYL family | -0.8408 |
|  | TraesCS2A02G000400 | Protein phosphatase 2C | 2.4016 |
|  | TraesCS3B02G277900 | Protein phosphatase 2C | 2.0312 |
|  | TraesCS3A02G249000 | Protein phosphatase 2C | 1.9869 |
|  | TraesCS4D02G210900 | Protein phosphatase 2C | 1.7395 |
|  | TraesCS1A02G411200 | Protein phosphatase 2C | 1.5852 |
|  | TraesCS5B02G182000 | Protein phosphatase 2C | 1.5126 |
|  | TraesCS2B02G023600 | Protein phosphatase 2C | 1.4618 |
|  | TraesCS4A02G094300 | Protein phosphatase 2C | 1.3780 |
|  | TraesCS3D02G249000 | Protein phosphatase 2C | 1.1148 |
|  | TraesCS5A02G183600 | Protein phosphatase 2C | 1.0776 |
|  | TraesCS3A02G362200 | Protein phosphatase 2C | 1.0683 |
|  | TraesCS5D02G188600 | Protein phosphatase 2C | 1.0251 |
|  | TraesCS1A02G358600 | Protein phosphatase 2C | 1.0028 |
|  | TraesCS1B02G375100 | Protein phosphatase 2C | 0.6910 |
|  | TraesCS3B02G394600 | Protein phosphatase 2C | 0.6092 |
|  | TraesCS2B02G521800 | Serine/threonine-protein kinase SRK2 | 0.8945 |
|  | TraesCS3D02G371900 | ABA responsive element binding factor | 2.1472 |
|  | TraesCS3A02G378700 | ABA responsive element binding factor | 1.5214 |
| Sucrose synthesis | TraesCS4B02G167500 | Sucrose synthase | 1.8076 |
|  | TraesCS3A02G015500 | Sucrose-phosphate synthase | 0.8146 |
|  | TraesCSU02G044500 | Sucrose-phosphate synthase | 0.6443 |
| Starch decomposition | TraesCS5A02G513200 | Beta-amylase | 1.2404 |
|  | TraesCS4B02G344300 | Beta-amylase | 1.2347 |
