# Supplementary material for: Transcriptome analysis reveals the mechanism by which spraying diethyl aminoethyl hexanoate after anthesis regulates wheat grain filling
Source: BMC Plant Biol. 2019 Jul 19;19:327. doi: 10.1186/s12870-019-1925-5 (PMC6642493; doi:10.1186/s12870-019-1925-5)
Supplement: Supplementary file 6 — Table S3 List of selected genes for KEGG pathways in T6vsT0. (DOCX 15 kb) [file 12870_2019_1925_MOESM6_ESM.docx]

**Table S3.** List of selected genes for KEGG pathways in T6vsT0.

|  | Gene ID | KO | Log_2_(fold change) |
| --- | --- | --- | --- |
| ER-associated degradation | TraesCS3D02G351900 | heat shock 70kDa protein | 3.4357 |
|  | TraesCS1B02G294300 | heat shock 70kDa protein | 1.4584 |
|  | TraesCS1D02G284000 | heat shock 70kDa protein | 1.4567 |
|  | TraesCS2D02G033200 | molecular chaperone HtpG | 3.8580 |
|  | TraesCS2B02G047400 | molecular chaperone HtpG | 3.7237 |
|  | TraesCS2A02G033700 | molecular chaperone HtpG | 2.5696 |
|  | TraesCS7A02G242200 | molecular chaperone HtpG | 0.7906 |
|  | TraesCS5B02G428000 | DnaJ homolog subfamily A member 2 | 0.7832 |
|  | TraesCS4A02G226700 | HSP20 family protein | 5.6782 |
|  | TraesCS3B02G049900 | HSP20 family protein | 4.8655 |
|  | TraesCS6B02G210600 | HSP20 family protein | 4.3351 |
|  | TraesCS4D02G212300 | HSP20 family protein | 3.6108 |
|  | TraesCS4B02G211700 | HSP20 family protein | 3.1193 |
|  | TraesCS6B02G374100 | HSP20 family protein | 2.7099 |
|  | TraesCS3D02G115300 | HSP20 family protein | 2.4608 |
|  | TraesCS7B02G347100 | HSP20 family protein | 2.3957 |
|  | TraesCS6D02G322300 | HSP20 family protein | 2.0506 |
|  | TraesCS6A02G316200 | HSP20 family protein | 2.0122 |
|  | TraesCS6B02G346700 | HSP20 family protein | 1.9705 |
|  | TraesCS5A02G257700 | HSP20 family protein | 1.9325 |
|  | TraesCS4A02G092100 | HSP20 family protein | 1.5904 |
|  | TraesCS3B02G131200 | HSP20 family protein | 1.5640 |
|  | TraesCS4B02G212200 | HSP20 family protein | 1.4831 |
|  | TraesCS4D02G209700 | HSP20 family protein | 1.3087 |
|  | TraesCS4A02G095500 | HSP20 family protein | 1.2905 |
|  | TraesCS7B02G130900 | HSP20 family protein | 1.2384 |
|  | TraesCS4D02G213300 | HSP20 family protein | 1.0799 |
| Ubiquitin ligase complex | TraesCS3D02G351900 | heat shock 70kDa protein | 3.4357 |
|  | TraesCS1B02G294300 | heat shock 70kDa protein | 1.4584 |
|  | TraesCS1D02G284000 | heat shock 70kDa protein | 1.4567 |
|  | TraesCS5B02G428000 | DnaJ homolog subfamily A member 2 | 0.7832 |
| Protein recognition by luminal chaperones | TraesCS7B02G446900 | heat shock protein 90kDa beta | -0.8146 |
