# Supplementary material for: Transcriptome analysis reveals the mechanism by which spraying diethyl aminoethyl hexanoate after anthesis regulates wheat grain filling
Source: BMC Plant Biol. 2019 Jul 19;19:327. doi: 10.1186/s12870-019-1925-5 (PMC6642493; doi:10.1186/s12870-019-1925-5)
Supplement: Supplementary file 7 — Table S4 List of selected genes for KEGG pathways in S6vsS0. (DOCX 16 kb) [file 12870_2019_1925_MOESM7_ESM.docx]

**Table S4.** List of selected genes for KEGG pathways in S6vsS0.

|  | Gene ID | KO | Log_2_(fold change) |
| --- | --- | --- | --- |
| Starch synthesis | TraesCS7A02G549100 | 1,4-alpha-glucan branching enzyme | 0.8337 |
|  | TraesCS7D02G535400 | 1,4-alpha-glucan branching enzyme | 0.7135 |
|  | TraesCS7D02G535500 | 1,4-alpha-glucan branching enzyme | 0.5699 |
|  | TraesCS7B02G472500 | 1,4-alpha-glucan branching enzyme | 0.4541 |
| Translocon (Protein export) | TraesCS3D02G208200 | protein transport protein SEC61 subunit beta | 0.8097 |
|  | TraesCS3A02G207800 | protein transport protein SEC61 subunit beta | 0.6356 |
|  | TraesCS3B02G234900 | protein transport protein SEC61 subunit beta | 0.4872 |
| Protein targeting | TraesCS4A02G214200 | protein disulfide-isomerase | 0.8182 |
| ER-associated degradation | TraesCS4D02G206600 | heat shock 70kDa protein | 0.9301 |
|  | TraesCS4B02G205700 | heat shock 70kDa protein | 0.8817 |
|  | TraesCS4A02G098600 | heat shock 70kDa protein | 0.6077 |
|  | TraesCS4D02G140800 | heat shock 70kDa protein | 0.5897 |
|  | TraesCS1B02G151300 | heat shock 70kDa protein | 0.5493 |
|  | TraesCS1A02G133100 | heat shock 70kDa protein | 0.4852 |
|  | TraesCS1A02G285000 | heat shock 70kDa protein | 0.4414 |
|  | TraesCS2B02G047400 | molecular chaperone HtpG | 0.8660 |
|  | TraesCS2A02G033700 | molecular chaperone HtpG | 0.8110 |
|  | TraesCS2D02G033200 | molecular chaperone HtpG | 0.6719 |
|  | TraesCS4A02G095500 | hsp70-interacting protein | 0.8498 |
|  | TraesCS4B02G208900 | hsp70-interacting protein | 0.7518 |
|  | TraesCS4D02G209700 | hsp70-interacting protein | 0.7254 |
|  | TraesCS3D02G045600 | HSP20 family protein | 1.2053 |
|  | TraesCS4D02G212500 | HSP20 family protein | 1.1899 |
|  | TraesCS5B02G245700 | HSP20 family protein | 1.0641 |
|  | TraesCS3B02G131200 | HSP20 family protein | 1.0131 |
|  | TraesCS3B02G049800 | HSP20 family protein | 0.9982 |
|  | TraesCS3D02G045700 | HSP20 family protein | 0.9971 |
|  | TraesCS3A02G034000 | HSP20 family protein | 0.9791 |
|  | TraesCS3A02G113000 | HSP20 family protein | 0.9668 |
|  | TraesCS2A02G312900 | HSP20 family protein | 0.9473 |
|  | TraesCS3B02G049900 | HSP20 family protein | 0.9070 |
|  | TraesCS7B02G088700 | HSP20 family protein | 0.8805 |
|  | TraesCS3D02G045800 | HSP20 family protein | 0.8802 |
|  | TraesCS7A02G202200 | HSP20 family protein | 0.8714 |
|  | TraesCS2B02G329900 | HSP20 family protein | 0.8664 |
|  | TraesCS5D02G266000 | HSP20 family protein | 0.8605 |
|  | TraesCS2D02G311400 | HSP20 family protein | 0.8388 |
|  | TraesCS3D02G115100 | HSP20 family protein | 0.8355 |
|  | TraesCS5B02G257000 | HSP20 family protein | 0.8320 |
|  | TraesCS4B02G089800 | HSP20 family protein | 0.8104 |
|  | TraesCS3A02G113100 | HSP20 family protein | 0.8014 |
|  | TraesCSU02G194600 | HSP20 family protein | 0.7967 |
|  | TraesCS3B02G130500 | HSP20 family protein | 0.7900 |
|  | TraesCS4D02G086200 | HSP20 family protein | 0.7891 |
|  | TraesCS3D02G045500 | HSP20 family protein | 0.7685 |
|  | TraesCS3D02G115400 | HSP20 family protein | 0.7682 |
|  | TraesCS3B02G131100 | HSP20 family protein | 0.7682 |
|  | TraesCS4A02G226700 | HSP20 family protein | 0.7455 |
|  | TraesCS5A02G257700 | HSP20 family protein | 0.7442 |
|  | TraesCS4A02G068200 | HSP20 family protein | 0.7366 |
|  | TraesCS3D02G114900 | HSP20 family protein | 0.7183 |
|  | TraesCS4D02G145600 | HSP20 family protein | 0.7170 |
|  | TraesCS3B02G130300 | HSP20 family protein | 0.7133 |
|  | TraesCS3D02G115300 | HSP20 family protein | 0.7107 |
|  | TraesCS3D02G114800 | HSP20 family protein | 0.7065 |
|  | TraesCS3D02G115000 | HSP20 family protein | 0.6921 |
|  | TraesCS4B02G212200 | HSP20 family protein | 0.6911 |
|  | TraesCS7B02G347100 | HSP20 family protein | 0.6861 |
|  | TraesCS3A02G034500 | HSP20 family protein | 0.6424 |
|  | TraesCS4B02G212300 | HSP20 family protein | 0.6335 |
|  | TraesCS4A02G092700 | HSP20 family protein | 0.6226 |
|  | TraesCS4D02G213100 | HSP20 family protein | 0.5994 |
|  | TraesCS4A02G092100 | HSP20 family protein | 0.5954 |
|  | TraesCS7D02G232600 | HSP20 family protein | 0.5367 |
|  | TraesCS7D02G185600 | HSP20 family protein | 0.5188 |
|  | TraesCS7D02G464800 | ubiquitin conjugation factor E4 | 0.5072 |
| Ubiquitin ligase complex | TraesCS5A02G331300 | S-phase kinase-associated protein | 0.7406 |
|  | TraesCS5B02G331700 | S-phase kinase-associated protein | 0.7259 |
|  | TraesCS4D02G206600 | heat shock 70kDa protein | 0.9301 |
|  | TraesCS4B02G205700 | heat shock 70kDa protein | 0.8817 |
|  | TraesCS4D02G140800 | heat shock 70kDa protein | 0.5897 |
|  | TraesCS1B02G151300 | heat shock 70kDa protein | 0.5493 |
|  | TraesCS1A02G133100 | heat shock 70kDa protein | 0.4852 |
|  | TraesCS1A02G285000 | heat shock 70kDa protein | 0.4414 |
