# Supplementary material for: Transcriptome analysis reveals the mechanism by which spraying diethyl aminoethyl hexanoate after anthesis regulates wheat grain filling
Source: BMC Plant Biol. 2019 Jul 19;19:327. doi: 10.1186/s12870-019-1925-5 (PMC6642493; doi:10.1186/s12870-019-1925-5)
Supplement: Supplementary file 8 — Table S5 Primers used for qRT-PCR. (DOCX 14 kb) [file 12870_2019_1925_MOESM8_ESM.docx]

**Table S5.** Primers used for qRT-PCR.

| Primer | Sequence (5' to 3') |
| --- | --- |
| Actin-F | CGAAGCGACATACAATTCCATC |
| Actin-R | GAACCTCCACTGAGAACAACAT |
| TraesCS3B02G277900-F | CTGGCAGGTGGTTGGAGGAAT |
| TraesCS3B02G277900-R | GCAATAGGCTCAGGACGGATCT |
| TraesCS3A02G015500-F | GTGCCAATGGTCTTCACAGGTC |
| TraesCS3A02G015500-R | TCAGCCTCAATCCGTCGCATTA |
| TraesCS4A02G214200-F | CCCTGACAACCATCCTTACCTCT |
| TraesCS4A02G214200-R | CCTTGCCACTGAACTCCTCTACA |
| TraesCS2A02G033700-F | GCTGCTCTCGCTCATCATCAAC |
| TraesCS2A02G033700-R | TGCTCTTGTCTGTCAGGCTCTC |
| TraesCS4B02G211700-F | ACAAGGAGCAGGAGGAGAAGAC |
| TraesCS4B02G211700-R | TGACGGTGAGCACGCTGTT |
| TraesCS1D02G284000-F | CGGCATCCTGAACGTGTCTG |
| TraesCS1D02G284000-R | TCTTCTTGTGCTCCTCGTCCTC |
